# The past and future of neuropsychological assessment: Back to the origins to face digital technological changes

**DOI:** 10.1007/s10072-026-09263-2

**Published:** 2026-07-24

**Authors:** Marina Gasparini, Nicola Vanacore, Valentina Moro

**Affiliations:** 1 Cognitive and Language Rehabilitation Center “Sinapsy”, Rome, Italy; 2 National Health Institute, Roma, Italy; 3 Department Human Sciences, University of Verona, Via Lungadige Porta Vittoria, 17, Verona, Italy; 4 IRCSS Sacro Cuore – Don Calabria, Negrar, Verona, Italy

**Keywords:** Neuropsychological Assessment Procedures, Artificial Intelligence in Neuropsychology, Assessment of Dementia, Fixed and Flexible neuropsychological batteries

## Abstract

**Background:**

The procedures used for the neuropsychological assessment of patients with dementia have been undergoing significant transformations relating to technological developments and the diffusion of artificial intelligence tools.

**Objectives:**

The integration of a theoretical position and a practical approach is needed in clinical contexts so that newly available tools can enhance the fundamental principles underlying neuropsychological assessment.

**Methods:**

Following a historical review of the various approaches to neuropsychological assessment, an analysis of the risks associated with the introduction of digital techniques, when not supervised by expert clinicians, is presented. The need to integrate new technologies in a comprehensive assessment involving clinical observation, ad-hoc tests, and other sources of information such as neuroimaging and neurophysiological data is discussed.

**Results:**

The integration of new technologies with a clinical, personalised approach to the patient represents a challenge for neuropsychology that deserves ad-hoc research and discussion.

## Introduction

During and after the SARS-CoV-2 (Covid-19) pandemic, medical practice methods changed as a result of social isolation, and these were accompanied by an increasing interest in tele-medicine. There have also been a number of studies published on the use of virtual environments in cognitive assessments in Neuropsychology [1, 2].

Tele-neuropsychology has been around for some time [3, 4]. Telephone, videoconference, and web-based instruments have been used for brief screenings, clinical monitoring and post-intervention follow-ups. Data shows that although it cannot be assumed that remote and in-person assessments are equivalents, these instruments are often extremely valid [2]. Nevertheless, a risk of systematic errors has been reported, for example when the MMSE is carried out by means of a video conference, with an overestimation of deficits in patients suffering from dementia [5].

Evaluation tasks using virtual environments were until recent years administered by specialist clinicians with experience of diagnosing cognitive diseases, and work groups (e.g. https://tela20.net/) were involved in the monitoring of telemedicine practices, with national guidelines to control these practices (in Italy by the Italian National Healthcare System, DL n. 179/2012; DM n. 256/2022). To prevent the misuse of computerised tests in clinical settings, the American Academy of Clinical Neuropsychology and the National Academy of Neuropsychology also published a joint position paper [6] and made some position statements on key issues relevant to the development and use of digital devices in neuropsychological testing.

More recently, thanks to the widespread use of Artificial Intelligence (AI), online testing in “blind” conditions have become the latest phenomenon for cognitive evaluation. During the administration of a test, there is no contact between the person being tested and the psychologist who only intervenes at the end of the process to assess the scores. An example in Italy involves remote neuropsychological assessments (NPA) which aim to identify cognitive problems in older people. The tests are usually relatively quick to administer (about 20 min) and can be done autonomously by an individual on a device in an affiliated pharmacy. The final report is written by a neuropsychologist and received after a few days (https://www.testperditamemoria.it/) or directly generated by an AI algorithm (e.g. https://www.cognitivetest.me/it)the. Mobile adaptations of standard neuropsychological tests are nowadays available for self-administration as well (e.g. the Stroop test [7]; the Trail Making Test [8]; the Symbol Digit Modalities Test [9]) and there are some validation studies of these smartphone-based versions of assessment batteries [10].

We see a radical transformation taking place. Fast moving technological developments associated with a progressive reduction in the costs of accessing technology, mean that clinicians and researchers in the field of Neuropsychology (as well as others involved in healthcare decision-making) need to now consider the potential effects of these changes. This position paper presents some issues regarding what the authors consider to be the essential aspects of NPA in the context of physiological aging and dementia, in order to ensure them in future clinical contexts. Without denying the usefulness of digital technologies in certain neuropsychological contexts, the authors believe it is worthwhile to keep the debate open regarding the risks of over-reliance on this approach in cognitive assessment, particularly where there is a lack of interaction between the patient and the neuropsychologist. After a short revision of the definition and the history of neuropsychology, the main methodological and procedural issues associated with NPA will be addressed. Some suggestions regarding the possibility of integrating the use of computerized, technologically advanced tools in clinical settings will conclude the article.

## The core of neuropsychology

By definition, “*Neuropsychology is an applied science concerned with the behavioral expression of brain dysfunction”* [11].

It is complex by nature and as such necessitates an interdisciplinary approach to cognition. Neuropsychology is also complex, due to the fact that the brain is plastic and constantly changes as a result not only of the biological processes it is subject to, for example physical maturing, aging or diseases, but also due to its operating mode which is profoundly grounded in the body and in interactions with the environment [12].

The neuropsychological approach necessarily correlates cognitive function deficits with changes in the central nervous system structures and functioning. This “anatomo-functional correlation” has been a key feature of Neuropsychology since its origins, but it was first systematized by Luria with his concept of “syndrome analysis” [13]. In the nineteenth century, there were debates between those with a localization approach (that is, the brain is a structure with localised functions), and a holistic approach which considered the whole brain to be involved in all body functions. Luria, however, overcame this debate and suggested the existence of systems underlying mental functions.

He made two main assumptions. The first was that complex cognitive functions are not located in discrete brain areas but are rather organised into functional systems which are composed of structures that are reciprocally connected or which work in synchrony. This means that different networks contribute to the same cognitive function, and a direct correlation between a cognitive impairment and a specific brain localisation is therefore problematic. The second assumption was that cognitive functions are always the result of an interaction between biology, individual experiences, social interactions and environment. These intuitions have since been confirmed in a number of studies on neural plasticity and brain damaged patients which have demonstrated the crucial role of white matter disconnections and functional connectivity in neuropsychological diseases [14]. Nevertheless, this means NPAs face considerable challenges due to the complexities of taking into account all of these dimensions and the need to grapple with concepts regarding “norms” and “individual variability”.

Luria preferred an approach which qualified rather than quantified deficits [15]. He was extremely critical of the standardised methods being used in the United States at that time [16], for example, the so called “fixed battery approach” pioneered by Halstead and Reitan [17] which used systematic procedures and case comparisons to interpret individual test scores [18]. Nevertheless, a seminal contribution to the development of validated tools was made by Benton and the Iowa-Benton School of Neuropsychology in the USA [19]. They proposed integrating standardised measures in a flexible way. The Boston Process Approach [20] also took on this perspective, focusing on the strategies a patient used to perform a task (that is, how they reached their answers) rather than looking the quantitative scores. In this way, the objective for Neuropsychologists became to see if there were any residual abilities beyond the patient’s deficits. In a similar way to Luria, the idea was for an NPA to not only quantify individual deficits but to define a cognitive profile, with data integrated with neuroimaging to provide diagnostic clues to etiology [21].

Nowadays, the main criticism toward a traditional approach concerns a failure to incorporate the advances in modern programs of data processing and technology into clinical practice (“Neuropsychology 3.0”) [22]. Novelties involve the introduction of computerised adaptive testing, web-based assessments and bio-informatic strategies using platforms on mobile phones, wearables (equipped with sensors and specific software), and in general making use of the “internet-of-things”, in order to collect and send data to processing systems.

Advances in technology offer previously unimaginable opportunities to collect information and monitor patients. Instruments for Ecological Momentary Assessment (smartphone or wearable technologies) make it possible to record repeated measures in an individual (e.g. fatigue, physiological parameters) in various different moments and in real-world environments [23]. Passive smartphone sensors may be useful to collect data on the frequency of symptoms, such as tremors, involuntary movements [24, 25] or other sensorimotor parameters. In Smart homes, various automated electronic systems are integrated into the patient’s home to record motor activity (e.g. smart carpets and pressure mats for gait; automated pillbox for tracking medication adherence [26]). However, these are tools for monitoring behaviour, which is different — though possibly complementary — to a neuropsychological assessment.

Within this framework, the main question is whether and how NPA can benefit from the advantages offered by technological developments without losing its specificity.

## Methodological issues: What can or cannot neuropsychological tests say?

In the past, NPAs mainly focused on diagnostic processes relating to focal syndromes, but nowadays they represent an additional support in various clinical conditions and can be used as a tool to measure the effectiveness of interventions, as well as providing a source of information for medico-legal or insurance purposes. Even in cases where there is more than one target to be investigated, the main reason why a patient is undergoing the examination should always be considered a priority, as this will guide the choice of the most appropriate tests.

The fundamental guidelines were defined by Lezak [11] in his seminal work: “…*tailor the examination to the patients’ needs*,* abilities*,* and limitations”* (p. 98). From this perspective, an NPA is conceived to be an “investigative” process in which explanatory hypotheses are generated to elucidate certain symptoms and then tested by means of specific tasks.

This becomes even more critical when assessing older adults and when an NPA is needed in order to identify potential signs of dementia. In this context, there are two main goals: (1) to differentiate between normal age-related changes and symptoms suggestive of a disease and (2) to identify a pattern of core deficits supporting various different diagnostic hypotheses. Italian guidelines for the diagnosis and management of dementia and Mild Cognitive Impairment (MCI) have identified NPAs as a core step within the diagnostic process of both Dementia (Recommendations n. 10: *strong*,* positive*) and MCI (Recommendation n. 28: *strong*,* positive*) (https://www.iss.it/-/snlg-diagnosi-e-trattamento-delle-demenze).

In accordance with these recommendations, most Italian Centres for Cognitive Disorders and Dementia (CCDD) have adopted a “fixed-flexible” approach using a Minimum Core Test (MCT) [27]. The MCT is based on scientific recommendations, which express the need to explore major cognitive functions as good predictors of any progression from subtle impairments to dementia [28, 29]. It is composed of a relatively small, fixed battery of tests, which will be supplemented by additional flexible measures, based on the neuropsychological diagnostic hypotheses arising during the assessment or if there are specific aims.

Again in the context of dementia, the evaluation is further refined when a patient’s decision-making abilities are assessed. Due to the ethical implications of the assessment, an in-depth search for residual abilities becomes crucial in order to determine whether there is any marginal capacity, as well as the patient’s self-awareness, their emotional reactions and the quality of their support network (http://www.regioni.it/newsletter/n-3900/del-10-08-2020/raccomandazioni-per-la-governance-e-la-clinica-nel-settore-delle-demenze-21590/).

Ultimately, with regard to elderly patients, NPAs need to take into account certain factors: (i) many of the tests used for adults are too difficult for elderly patients; (ii) although in western countries the number of adults over 85 is increasing, there are very few normative values for this age group; and (iii) fatigue occurs sooner in older patients, and an adaptation of assessment sessions is needed (e.g. with breaks and/or multiple short sessions). Finally, (iv) for people who are well-educated high performers, a normal score for a task might in their case represent a decline and this needs to be taken into consideration [30]. For these reasons, and considering that normal aging is in itself associated with a cognitive decline, caution is needed when fixed batteries of tests are used.

However, emphasising qualitative aspects does not mean that it is not worth quantifying an individual’s performance with specific scores. Whatever the aim of an assessment, the use of validated tests is, in fact, mandatory in NPAs to allow a comparison with normative data. Validation ensures that the test has adequate sensibility and specificity for assessing the cognitive function it was designed to measure. However, due to the distributed brain organization of the cognitive functions, even simple tasks activate diffuse neuroanatomical networks involving multiple functions (e.g. the token test involves language, attention, colour and form discrimination, as well as working memory). For this reason, an impairment in one function may affect the patient’s performance in tests devised to assess another function. On this basis, it is unrealistic to expect a test to be so selective for a single function and only consider a single test as a “marker” for diagnosis. The relationship between a test score and a cognitive deficit is not one to one; rather it is the result of an NPA taken as a whole that provides a pattern indicating impairments. This in turn allows clinicians to advance hypotheses about the involvement of particular anatomical locations and etiology [31].

It is also the case that the accuracy of measurements may not always be perfect. Firstly, there are no normative data for all of the variables potentially affecting performance (e.g. social environment, medications, general health condition or quality of sleep). Another aspect which can be challenging concerns the potential impact of the varying cultural backgrounds and languages of the patients, and this cannot be dealt with simply by translating as the tests are grounded in the culture in which they were devised (32). Again, the tasks often lack ecological significance and a patient’s ability to solve problems in their everyday context does not necessarily predict their performance in a clinical setting, and vice-versa (32). Secondly, when devising a test, it can be challenging to determine the degree of difficulty. Crucially, there have been improvements in population’s performance in tests (the Flynn Effect) and if the normative values are not updated, there is a greater likelihood that test scores will overestimate or underestimate real abilities (33) and this needs to be taken into consideration when results are interpreted. It may consequently be very difficult to compare the results from different tests based exclusively on quantitative scores.

Another methodological point concerns intrinsic and extrinsic factors which potentially affect performance and procedural aspects (see point 4). Some intrinsic factors, such as demographic variables (age, gender and education), can be controlled for the most part by means of a correction score – a statistical solution which has been used in Italian studies since 1988 [34]. Other aspects, such as socio-cultural issues, the cognitive effects of drugs (e.g. anti-epileptic), general health conditions (e.g. depression, sensory decline or infections), all of which may affect attention, concentration and memory can be only taken into consideration in a clinical face-to-face assessment. The same goes for many extrinsic factors that can be mitigated during in person testing: the length of the tasks, the sequence in which they are administered and their cognitive load, as well as ensuring a distraction-free environment.

## What is the diagnostic value of NPAs?

Given the methodological and procedural issues outlined above, what is the point of carrying out such a time-consuming and demanding examination? Among the many possible responses, the concept of incremental validity plays a key role. In 1996, the American Psychological Association’s Board of Professional Affairs established a Psychological Assessment Work Group to collect evidence on the efficacy of NPAs in clinical practices. Recommendations were released in two reports [35, 36]. In a meta-analysis of the literature [37], the same authors quantified the strength of the association between a predictor test and the variable object of investigation. They concluded that although there are varying degrees of validity in psychological tests (ranging from uninformative to strongly predictive results for a given criterion), validity coefficients for many tests are similar to those observed in medical tests. Neuropsychological tests correlated highly with the differentiation between people suffering from dementia and the controls (0.68), as did the tests assessing long-term verbal memory in the differentiation between dementia and depression (0.61). These values were slightly higher than those of MRI in the cases of dementia as compared to the controls (0.57). The authors thus concluded that “*psychological test validity is strong and compelling*” but also that “a *multimethod assessment battery provides a structured means for skilled clinicians to maximize the validity of individualized assessments*” (p.128) [37].

In terms of validity, the potential comparability between psychological and medical tests is not irrelevant to our debate as this leads to a notion of “*incremental validity*” with regard to NPAs. According to the definition established by Haynes and Lench [38], incremental validity is *the degree to which a measure explains or predicts some phenomena of interest*,* relative to other measures.* In effect, neuropsychological tests have been showing high levels of validity in predictive contexts since the 1980 s [39], and still provide a strong contribution in terms of diagnostic clarification and predictions relating to disease progression, particularly in differentiating MCI from normal aging and conversion from MCI to dementia [40]. This contribution is even more striking when an NPA encompasses a broad, multi-method evaluation of a patient that involves: (i) one or more interviews with the patient and their relatives; (ii) a review of clinical data; (iii) general screening measures and (iv) standardised face-to-face tests for specific cognitive domains chosen to be consistent with the patient’s ability [41].

The whole process helps to generate hypotheses about the nature of the disturbance, and to create a clinical picture with the lowest risk of false positives or negatives [33, 42, 43] . To support a causative diagnosis adequately, an accurate NPA should ultimately provide three diagnostic levels.

- *Descriptive diagnosis*: the description and integration of symptoms and signs. At this level, symptoms are only described but not explained. For example, a memory impairment is reported, but not how it occurs.
- *Functional diagnosis*: the definition of the cognitive functions and their subprocesses that are compromised. For example, a memory deficit may depend on a disorder either in the processes relating to the recall, storage or retrieval of information, or in some cases to attentional deficits. A number of different cognitive profiles emerge, typical of either cortical or subcortical pathologies.
- *Neuroanatomical diagnosis*: a hypothesis concerning the cerebral networks associated with the cognitive deficits. For example, the anterograde memory deficit - typical of cortical dementia - is functionally connected to the temporo-mesial (hippocampal) networks. Conversely, a deficit in recall processes depends more on the activity of the prefrontal network.

The integration of these three levels results in a fully informative NPA, which requires a high degree of multiple skills and the integration of other team members.

## Conclusions: Looking for a balance between technological devices and NPA

With critical healthcare changes over the past decades, there has also been a gradual decline in the use of time-consuming procedures, and a tendency to formalise standard, faster test procedures in NPAs. The aim of this paper is not to discuss the *pros* and *cons* of digital technology in the NPA, a topic that has already been extensively covered in many reviews [44–46], but rather to preserve the cultural and scientific importance of the NPA, and to reaffirm the key role of the human and empathetic relationship between clinician and patient.

What emerges from the previous considerations is that carrying out an NPA is a complex process, requiring a body of knowledge: a clinical competence to understand how a neurological disorder or other conditions may affect cognition and behaviour; a statistical background which makes it possible to understand the strengths and limitations of the various tests; and, last but not least, the capacity to communicate effectively with patients and their families. Making final diagnostic judgments may be challenging, particularly in the case of very mild symptoms [36] as there is a high variability in individual abilities in healthy people due to both inter-individual factors (genetics, age, environment) and intra-individual variables (daily fluctuations in cognitive performance) [47]. These are the main reasons why the process is by nature time-consuming and requires highly professional standards.

Nowadays, criticisms of classic approaches to NPAs mainly regard difficulties in incorporating advances in neuroscience, modern psychometric methods and technology into clinical practices [22]. Although NPAs have undergone changes in procedures and data processing as computerised programs have become available, the implementation of AI is accelerating this process.

There are now new opportunities in terms of processing large amounts of data, as well analysing large amount of incoming information from various different geographical, cultural and social contexts. Digital tools allow the extraction of high-dimensional data (e.g., response times, intra-individual variability, error dynamics), enabling a more refined analysis of cognitive processes and performance. AI also makes it possible to “simulate” behaviours and performances based on data and to develop models relating to behaviour and cognitive processes. But AI is not intelligent in itself and, at least to date, cannot perceive and consider the nuances underlying an individual patient’s performance, either in terms of cognitive strategies and the quality of errors made or in terms of emotional responses. When AI is used, data are analysed in statistical terms but there is no subsequent interpretation. When a diagnostic hypothesis is advanced, this is based on statistical probabilities. The lack of patient/examiner interaction, typical of computerised approaches, means that a patient’s behaviour and emotions during testing are not recorded [48].

A recent comparison of in-person and virtual cognitive assessments aimed at diagnosing dementia or MCI by means of widely used screening tests (i.e. MMSE and MoCA) [1] showed that the main barriers related to technological approaches concern the fact that it is impossible to record nonverbal signs which might potentially suggest a cognitive impairment, or planning breaks when necessary. Errors may be produced also because patients are unfamiliar with the technology. Furthermore, the abovementioned study stresses the importance of supportive or corrective feedback during NPAs, and concludes that no computerised program, no matter how sophisticated, is capable of providing these *“uniquely human” capacities*.

What might be a reasonable compromise between the use of new technologies and the need for reliability in diagnostic procedures? Thanks to their fast administration and automatic scoring, digital cognitive tests could be properly deployed in primary medical care for neurological screenings, to identify people at risk for neurodegenerative illness. However, one major point of attention involves their psychometric properties, as some studies showed low-moderate levels of reliability and validity (for a review, see Harris, 2014 [49]). More importantly, most computerised paradigms validated for clinical use are adaptations of the original paper-and-pencil tests, and the lack of appropriate norms or validation precludes their usage [50]. In fact, computerised adaptive testing is potentially very useful, in particular in rehabilitation settings, to assess the patients’ recovery. This may contribute towards the operationalisation of the principle of individualised assessments in a systematic, scalable way, but only when the same procedures of adaptation to the patients’ performance are validated. An integration of in-person with remote testing may also present some practical advantages. Remote assessment may be recommended for people who find it difficult to reach specialist centres, or as a way of collecting general, preliminary data or for specific follow-up assessments for patients who are already known to an examiner. Furthermore, when computerised techniques are used, and data are analysed by means of algorithms, these should be always checked by clinicians and integrated with the relevant clinical information. This specific point was highlighted in the aforementioned joint position statement [6]: the interpretation of the results “*requires the same specialized training and expertise in clinical neuropsychology as the interpretation of examiner-administered neuropsychological tests”* (pg. 365).

Finally, the combination of large databases and the use of AI will enable clinicians to gain knowledge and update normative data. However, the potential effects of uploading and elaborating data need to be borne in mind by clinicians and public health policymakers. The issue of ethics in the digital world is still under discussion, with a view to ensuring socially acceptable technologies and mitigating the risks associated with digital innovation. Ecologically momentary assessment, passive smartphone sensors or wearable devices may represent a significant advancement in patient assessment. Recording physiological parameters and subjective symptoms during the day (such as fatigue, perceived decrease in attention or pain) allows the examiner to better understand reported symptoms and relate them to everyday life situations. However, in addition to interface usability and security parameters, digital data collection requires special precautions to safeguard the right to privacy regarding genetic, biometric and health data.

In our opinion, technological developments will continue to offer new opportunities, but we must not underestimate the risk of entrusting the entire NPA to digital algorithms, first and foremost due to the necessity to predict daily life abilities from specific data sources [51]. Neuropsychologists in both social and healthcare systems nowadays have certain challenges to face: how to make the best use of technology while keeping it within a person-centred approach, and - as required by the guidelines – the need to develop a high level of skills.
